# Invasive Group B Streptococcal Disease in Two Pediatric Patients with Systemic Lupus Erythematosus

**DOI:** 10.1155/2013/896014

**Published:** 2013-04-04

**Authors:** Dongning Wu, Kenneth Bromberg, Roberto Jodorkovsky

**Affiliations:** ^1^Department of Pediatrics, The Brooklyn Hospital Center, 121 DeKalb Avenue, Brooklyn, NY 11201, USA; ^2^Department of Pediatrics, St. Joseph's Hospital, Patterson, NJ 07503, USA

## Abstract

Systemic lupus erythematosus (SLE) is an autoimmune disease associated with high morbidity and mortality, often caused by infection. We report two patients with SLE who were treated with steroids and immunosuppressive medication and then developed invasive Group B *Streptococcus* (GBS) infections. While GBS infection is rare in the nonneonatal pediatric age group, GBS should be considered when treating SLE patients presenting with signs of infection.

## 1. Introduction

Systemic lupus erythematosus (SLE) is an autoimmune inflammatory disease which can cause severe acute and chronic multiorgan damage and death. Treatment with corticosteroids and other immune-suppressive drugs has improved the 5-year-survival rate of SLE from 50% in the mid-fifties to over 90% in the last decade. However, this treatment is still associated with serious complications, including severe infections. Invasive secondary infections can be responsible for approximately 25% of SLE related deaths [1, 2].

Invasive Group B *Streptococcus* (GBS) infection is a leading cause of illness and death among young infants and pregnant women. Additionally, several recent reports suggest that the incidence of GBS disease has also been increasing among older adults with underlying medical conditions affecting their immune system [3]. There is only one published report retrievable by a Medline search (using SLE and GBS) documenting a serious GBS infection involving an 18-year-old adolescent with SLE [4].

We report two cases of severe invasive GBS infection in two nonpregnant adolescents being treated for SLE nephritis.

## 2. Case 1

A 17-year-old African American female was diagnosed with class IV lupus nephritis 2 months prior to her presentation with a one-day history of progressive left flank pain, diffuse abdominal pain, fever, and nonbilious, nonbloody vomiting. The patient was being treated with prednisone, 60 mg/day, mycophenolate mofetil, 2000 mg/day, enalapril, 20 mg bid, losartan, 25 mg bid, and hydroxychloroquine, 400 mg/day. Physical examination was remarkable for an oral temperature of 38.5°C, HR 128/min, blood pressure 126/84 mmHg, a tired appearance, and significant left lower quadrant tenderness and rebound pain. Laboratory tests showed WBC 21.6 × 10^3^/mm^3^ with 90% neutrophils, normal renal function and electrolytes, total proteins 4.4 g/dL, serum albumin 3.0 g/dL, urine protein/creatinine 10.44, C3 84 mg/dL (normal 82–193), and C4 17.6 mg/mL (normal 15–57). Abdominal ultrasound showed scattered peritoneal fluid deemed difficult to tap due to its small volume. The patient was diagnosed with primary peritonitis and started on ceftriaxone and vancomycin. She experienced significant and swift improvement of her symptoms. Blood and urine cultures grew penicillin sensitive Group B *Streptococcus*. After completing treatment with IV penicillin the patient was discharged home in good clinical condition. 

## 3. Case 2

A 16-year-old female with class V lupus nephritis presented with progressive bilateral leg swelling and pain for one week and a fever for 3 days. The lupus nephritis had been diagnosed one year before and initially responded well to induction treatment with high dose steroids and mycophenolate mofetil. The patient experienced intermittent exacerbations of her nephrotic range proteinuria due to poor adherence to the treatment. On admission, her medication included prednisone, 60 mg qd, mycophenolate mofetil, 1.5 gm bid, hydroxychloroquine, 200 mg bid, enalapril, 20 mg bid, losartan, 50 mg bid, and lasix as needed. Physical examination was significant for toxic appearance, oral temperature of 38°C, blood pressure 88/52 mmHg, heart rate 168/min, prolonged capillary refill, generalized edema, and bluish discoloration and skin maceration on the lower extremities. Laboratory data showed WBC 3.1 × 10^3^/mm^3^, hemoglobin 9.6 g/dL, PTT 51.5 sec, PT 15.5 sec, INR 1.6, D Dimer 14.91 mg/L (normal < 1.2), fibrinogen 588 mg/L (normal 205–410), C3 39 mg/dL, C4 7.5 mg/dL, creatinine 1.6 mg/dL, total proteins 3.7 g/dL, and serum albumin 1.3 g/dL. A blood culture grew Group B *Streptococcus*. The patient was treated with vigorous fluid resuscitation, vasopressors, vancomycin and cefepime and continuous venovenous hemofiltration, and fasciotomy of the lower extremities. Therapy was changed to IV penicillin based on the identification of the organism. After several weeks, the patient recovered and was discharged home with a normal kidney function, but persistent heavy proteinuria.

## 4. Discussion

SLE is an autoimmune disease manifesting with a wide clinical spectrum ranging from single cutaneous manifestations to multiorgan failure. Secondary infections can be a leading cause of morbidity and mortality in SLE patients. Skin and soft tissue are the most common sites of infections followed by respiratory tract, bacteremia, urinary tract, gastrointestinal tract, peritoneum, bone, and joints. The most common bacterial infections have been attributed to *Streptococcus pneumoniae* and *Neisseria meningitides* [2]. Immunologic dysfunction predisposes SLE patients to develop potentially life-threatening infections. These abnormalities include reduced complement and immunoglobulin levels, functional asplenia, defective immune complexes clearance, and chemotaxis and phagocytosis defects. These abnormalities can be caused by both the lupus and its treatment with corticosteroids and other immunosuppressant agents [2]. In our two cases, both patients were being treated with prednisone. The low complement levels in case 2 are consistent with lupus activity and consistent with the patient being immunocompromised. The complement level in case 1 was tested seven days after starting therapy. The normal C3 and C4 levels correlated with patient's good response to antibiotics treatments. They may well have been abnormal at the time of presentation.

Primary bacterial peritonitis is a well-recognized complication of patients with ascites secondary to cirrhosis and the nephrotic syndrome. The infecting bacteria are mainly gastrointestinal tract normal flora, such as *Escherichia coli* and *Streptococcus pneumoniae*. There are very few reports on spontaneous peritonitis due to GBS [5].

Necrotizing fasciitis is an infection of the deeper layers of skin and subcutaneous tissue. It is a progressive life-threatening disease and has broad clinical presentation ranging from pain, erythema, and cellulitis to severe sepsis and multiorgan failure. Group A beta hemolytic streptococci are the most common cause of necrotizing fasciitis [6]. Several studies have reported necrotizing fasciitis associated with *Streptococcus pneumoniae* in SLE patients. The reports on GBS necrotizing fasciitis are generally limited to the adult population [7]. There are occasional single cases in large series that mention noninfant children [8].

GBS is a Gram-positive organism characterized by the presence of Group B Lancefield carbohydrate cell wall antigen and specific polysaccharide capsules that designate the serotype. GBS is a leading cause of illness and death among infants and of infection in pregnant women. Several studies suggested that the incidence of GBS disease is also increasing in nonpregnant adults, especially among older adults and among those with underlying medical conditions [3]. It increased by 32% during the period 1999–2005, reaching 7.9 per 100,000 among nonpregnant adults in 2005. In these data only 90 noninfant children (age 1–15) of 14,573 total patents (0.6%) were identified with invasive GBS disease. That is why we say that this is an uncommon infection in the noninfant pediatric age range (although our two patients were just on the cusp on this range) [9].

GBS infections frequently present with skin and soft tissue infections, bacteremia, pneumonia, and osteomyelitis. The main underlying medical conditions that predispose patients to develop invasive GBS are diabetes, atherosclerotic cardiovascular disease, heart failure, and cancer. GBS infection emerged as an important cause of morbidity and mortality in immune-compromised nonpregnant adults and raised more awareness and concerns in adult population [3]. Our two cases underscore the importance of early recognition, aggressive treatment, and possible prevention of invasive GBS disease in nonpregnant teenagers with SLE.

GBS are susceptible to penicillin, which is the antimicrobial of choice in treating affected patients. The most prevalent serotypes of invasive GBS isolated are Ia, Ib, II, III, and V among ten serotypes [3]. In our cases, the serotypes of GBS were not typed. Since studies have shown that pneumococcal vaccinations are safe and generally effective in SLE patients despite their impaired immune response [10], the ongoing development of GBS multivalent polysaccharide-protein conjugate vaccines based on serotype-specific capsular polysaccharides could potentially prevent pediatric, adult, and pregnant-associated diseases. In the future, typing of pediatric GBS isolates might be of benefit to vaccine policy. In the meantime, identification of infection and appropriate therapy remains important in SLE.

## Abbreviations

SLE: Systemic lupus erythematosus
GBS: Group B *Streptococcus*.
